# Optimization of *Monascus purpureus* for Natural Food Pigments Production on Potato Wastes and Their Application in Ice Lolly

**DOI:** 10.3389/fmicb.2022.862080

**Published:** 2022-06-01

**Authors:** Hossam E. F. Abdel-Raheam, Sulaiman A. Alrumman, Samir I. Gadow, Mohamed H. El-Sayed, Dalia M. Hikal, Abd El-Latif Hesham, Maysa M. A. Ali

**Affiliations:** ^1^Department of Food Sciences, Faculty of Agriculture, Beni-Suef University, Beni Suef, Egypt; ^2^Biology Department, College of Science, King Khalid University, Abha, Saudi Arabia; ^3^Department of Agricultural Microbiology, National Research Centre, Agriculture and Biology Research Institute, Cairo, Egypt; ^4^Department of Botany and Microbiology, Faculty of Science, Al-Azhar University, Cairo, Egypt; ^5^Nutrition and Food Science, Home Economics Department, Faculty of Specific Education, Mansoura University, Mansoura, Egypt; ^6^Department of Genetics, Faculty of Agriculture, Beni-Suef University, Beni Suef, Egypt; ^7^Botany and Microbiology Department, Faculty of Science, Assuit University, Assuit, Egypt

**Keywords:** potato powder wastes, *Monascus purpureus*, pigment, response surface methodology, chips manufacturing wastewater, ice lolly

## Abstract

During potato chips manufacturing, large amounts of wastewater and potato powder wastes are produced. The wastewater obtained at washing after cutting the peeled potatoes into slices was analyzed, and a large quantity of organic compounds and minerals such as starch (1.69%), protein (1.5%), total carbohydrate (4.94%), reducing sugar (0.01%), ash (0.14%), crude fat (0.11%), Ca (28 mg/L), Mg (245 mg/L), Fe (45.5 mg/L), and Zn (6.5 mg/L) were recorded; these wastes could be considered as valuable by-products if used as a fermentation medium to increase the value of the subsequent products and to exceed the cost of reprocessing. In this study, we used wastewater and potato powder wastes as a growth medium for pigment and biomass production by *Monascus purpureus* (Went NRRL 1992). The response surface methodology was used to optimize total pigment and fungal biomass production. The influence of potato powder waste concentration, fermentation period, and peptone concentration on total pigment and biomass production was investigated using the Box-Behnken design method with 3-factors and 3-levels. The optimal production parameters were potato powder waste concentration of 7.81%, fermentation period of 12.82 days, and peptone concentration of 2.87%, which produced a maximum total pigment of 29.86 AU/ml that include, respectively, a maximum biomass weight of 0.126 g/ml and the yield of pigment of 236.98 AU/g biomass. The pigments produced were used as coloring agents for ice lolly. This study has revealed that the ice lolly preparations supplemented with these pigments received high acceptability. Finally, we recommend using wastewater and potato powder wastes for pigment and biomass production, which could reduce the cost of the pigment production process on an industrial scale in the future.

## Introduction

Horticulture production has significantly increased to 23.7 million tons of food per day during the last 50 years. Some portion of biomass from agriculture products generates waste, which may not be used as food (Duque-Acevedo et al., 2020). In addition, industrial production consumes a significant amount of soil and water (Aguilera et al., 2020).

According to Kot et al. (2020), there is an increased need for biotechnological methods for biorefinery of the industrial waste to use as ingredients of culture media for microorganisms. This provides the complete biodegradation of organic compounds and the production of a new product with the added value. Moreover, the use of waste products as medium components reduces the total production costs. The sustainability of industrial production becomes concerned with the recovery and reuse of their wastes as a resource within the cycle of the circular economy (Günerhan et al., 2020).

Nowadays, there are intensive interests in environmental protection, which become one of the priorities of international politics. The accumulation of industrial wastes plays a significant role in the degradation of the environment, especially waste products of the food industry, as they contain a large amount of organic substances. Pathak et al. (2018) reported that ~0.16 tons of solid waste are obtained per ton of processed potato.

**Figure 1:**
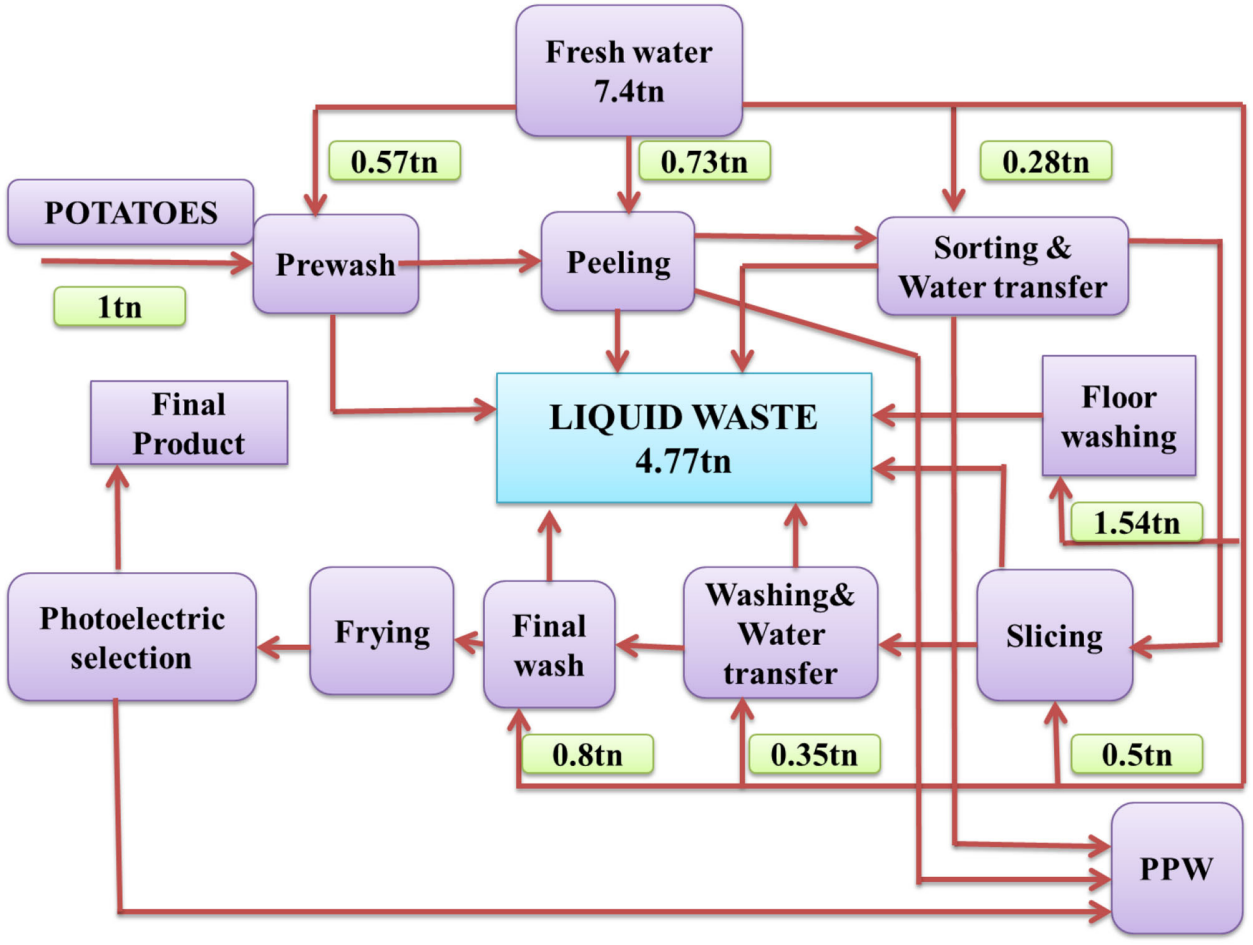
Diagram showing potato chips manufacturing (Arapoglou et al., 2010).

Large amounts of water are necessary to manufacture potato chips for washing, peeling, and blanching the raw material (Figure 1). Liquid waste generated from these operations is characterized by high starch content ranging from 20 to 25% g/L (Haung et al., 2003). After settling this water, large amounts of starch and solids are produced. These sediments could be used as the culture medium to produce valuable products by microorganisms at a low cost.

Recently, microbial pigments have received intensive attention because they are characterized by many medicinal properties, nutritional effects, and controllable and predictable yield (Global Food Color Market Research Report, 2021), mainly produced from agro-industrial by-products. *Monascus purpureus* is one of the most important producers of microbial pigments. Various food industry wastes were used to produce *Monascus* pigments in the literature. These are hydrolyzed rice straw (Liu et al., 2020), waste beer (Atalay et al., 2020), orange peels (Kantifedaki et al., 2018), chicken feather (Orak et al., 2018), sugarcane bagasse (Hilares et al., 2018), and potato powder (Sharmila et al., 2013). It produces a complex mixture of six chemically defined colored compounds of polyketide origin (Campoy et al., 2006). These compounds are rubropunctatine, monascorubrine (orange), rubropunctamine, monascorubramine (red), monascine, and ankaflavine (yellow) (Zhou et al., 2009). The red pigment is in high demand, especially for its use in meat products to substitute nitrites (Fabre et al., 1993). *Monascus* pigments are used for the natural coloring of oriental foodstuffs in Asian countries and the textile dyeing process (Santis et al., 2005; Velmurugan et al., 2010).

The *Monascus* pigments have been of increasing interest to the food industry as food colorants because *Monascus* products are extracellular and water-soluble making them easy to use. *Monascus* pigments applications include the increased red coloring in meat, fish, and ketchup (Hamano and Kilikian, 2006). It can also be added to dairy products such as fruit-flavored yogurt (Abdel-Raheam et al., 2019) and flavored milk (Gomah et al., 2017) for enhancing the natural color of the fruit. Similarly, it can be used for poultry products such as poultry salami (Mal'a et al., 2010) and for sweet products such as lollipops, jelly beans (Darwesh et al., 2020), and drops sweets (Abdel-Raheam et al., 2021). The results by previous investigators reported that food products gain more intense and stable color and improved organoleptic characteristics when *M. purpureus* pigments were used (Hamano and Kilikian, 2006 and Mal'a et al., 2010; Gomah et al., 2017; Abdel-Raheam et al., 2019, 2021; Darwesh et al., 2020).

Production of *Monascus* pigments by submerged fermentation was preferred instead of the solid-state process due to its easy control of process parameters, high productivity, large volume processing, reduced fermentation time, and cost (Zhou et al., 2014). It can also solve the problems of low income, capital intensive, time-consuming, and the necessity of large surface area in the case of solid fermentation. Moreover, this method is more appropriate for large-scale industrial production than solid-state production (Yuliana et al., 2017).

Response surface methodology RSM is successfully applied to optimize medium components and process parameters in various bioprocesses (Kalaivani and Rajasekaran, 2014). It is well-known that optimization using one parameter study is high cost and expends time, whereas response surface methodology (RSM) being reliable and easy to use reduces the number of experiment runs, and it also details the interaction effect between the variables involved in the process. Also, it is fast, low cost, and statistically acceptable for carrying out research in comparison to the usual one variable study (Ajdari et al., 2013). It was reported that response surface methodology (RSM) is a practical statistical approach for the optimization of pigment production (Sen et al., 2019).

This study investigates the biorefinery of potato chips manufacturing wastewater obtained at washing after potatoes cutting into slices for pigment production during submerged fermentation by *M. purpureus* Went NRRL 1992. It also aimed to optimize pigment and biomass production conditions by using RSM. Separation of red, orange, and yellow *Monascus* pigments from optimized culture and individually applying the separated pigments in ice lolly was also mainly investigated.

## Materials and Methods

### Wastes Used

First, potato chips manufacturing wastewater of cutting (slicing) and soaking was obtained from a potato chips factory at Assuit Governorate, Egypt.

Second, potato chips waste powder produced after settling wastewater and then dried under sunlight was packed in polyethylene pages at 4°C until use.

### Chemical Composition Analysis of Potato Chips Manufacturing Wastewater

The contents of crude protein, crude fat, ash, reducing sugar, and carbohydrate as a percentage (W/V) were determined using standard methods of the Association of Official Analytical Chemists (AOAC, 2005).

The mineral contents of potato chips manufacturing wastewater were determined by Perkin-Elmer Atomic Absorption Spectrophotometer 2,380 for calcium, iron, zinc, and magnesium. The determination was carried out in the Central Laboratory, Faculty of Agriculture, Assiut University, as described in AOAC (1995).

### Microorganism

The strain used in this study was *M. purpureus* Went NRRL 1992 obtained from Microbiological Resources Center (MIRCEN), Ain Shams University Cairo, Egypt. It was maintained on a Yeast Extract–Peptone–Dextrose (YEPD) agar medium (1% yeast extract, 2% peptone, and 2% dextrose) at 4°C and subcultured periodically every 3 weeks. This strain was tested for its ability to produce citrinin as mycotoxin and found to be nonproducing (Abdel-Raheam, 2016). Inoculum preparation *M. purpureus* Went NRRL 1992 was grown on YEPD slant at 30°C. To fully sporulated (6–8 days old) agar slope culture, 10 ml of sterile distilled water was added and the spores were scraped under strict aseptic conditions. The spores suspension obtained was used as inoculum (~7 × 10^5^ spores per ml) as described by Babitha et al. (2007).

### Fermentation Medium

Submerged cultures were carried out at a 250-ml Erlenmeyer flask with a working volume of 100 ml; the fermentation medium was composed mainly of potato chips manufacturing wastewater. Different concentrations of potato waste powder as carbon source and different peptone concentrations as nitrogen source were used as supplements for wastewater. Flask contents were mixed well, fermentation medium was adjusted at pH 6.5, and then autoclaved at 121°C for 15 min after cooling at room temperature; flasks were inoculated with 10 ml fungal spore suspension and incubated at 30°C in the dark for the different incubation periods.

### Optimization of Pigment and Biomass Production and Experimental Design

The optimum conditions for total pigment production and biomass of *M. purpureus* Went NRRL 1992 were evaluated using response surface methodology (RSM) provided by Design Expert Software (2011) with a standard tool known as Box-Behnken design (BBD) (Box and Behnken, 1960) for testing the effect of independent variables and their interaction. Three factors were chosen based on preliminary tests for this work: potato powder (A), fermentation period (B), and peptone concentration (C). Three levels of each factor were included (Table 1).

**Table 1:** Values of independent variables in the Box-Behnken design.

**Parameters**	**Units**	**Coded levels**		
**(independent variables)**		**−1**	**0**	**+1**
Potato wastes (A)	%	5	7.5	10
Fermentation period (B)	day	7	10	13
Peptone concentration (C)	%	2	3.5	5

The pigment production response and biomass are estimated by a model equation RSM second order as follows:


(1) $$\begin{array}{l} \text{Y }=\;{\text{b}}_{0}+{\text{b}}_{1}\text{A}+{\text{b}}_{2}\text{B}+{\text{b}}_{3}\text{C}+{\text{b}}_{12}\text{AB}+{\text{b}}_{13}\text{AC}+{\text{b}}_{23}\text{BC}\\ \;+\;{\text{b}}_{11}{\text{A}}^{2}+{\text{b}}_{22}{\text{B}}^{2}+{\text{b}}_{33}{\text{C}}^{2}. \end{array}$$

Where Y is the dependent variable, and A, B, and C are independent variables.

### Determination of Pigment

At the end of the fermentation process, the medium was filtered using Whatman No. 1 filter paper, and the mycelia were washed twice with distilled water. The extracellular pigments were determined at the filtrate, which was measured by using a UV visible spectrophotometer (Abilene 9400—SCHOTT Instruments, EU) at 400, 470, and 500 nm for yellow, orange, and red pigments, respectively (Carels and Shepherd, 1977; Lin et al., 1992; Orozco and Kilikian, 2008) (Equation 3).

The intracellular pigment was extracted from the washed mycelia by transferring (0.1 g) to a 50-ml Erlenmeyer flask and 10 ml of ethanol 95% was added. The suspension was allowed to stand at 30°C for 12 h (Tseng et al., 2000). The supernatant was then centrifuged (using Himac CR 22GII, Hitachi Koki Company Limited, Japan) at 4,000 rpm for 10 min. The absorbance of the clear solution was measured at 500, 470, and 400 nm using a UV visible spectrophotometer (Chen and Johns, 1993) (Equation 4). Extra- and intracellular pigments yield in absorbance unit (AU) per ml were calculated by using the following formula (Mekhael and Yousif, 2009) (Equation 2). The yield (AU/g) was defined as the total pigments produced by every gram of biomass.


(2) $${\text{AU}}_{\text{Total pigment}}\;=\;{\text{AU}}_{\text{Total extra pigment}}+\;{\text{AU}}_{\text{Total intra pigment}}$$

(3) $${\text{AU}}_{\text{Total extrapigment}}\;=\;{\text{AU}}_{\text{Extra}}\times \text{df}\;$$

(4) $${\text{AU}}_{\text{Total intrapigment}}\;=\;({\text{AU}}_{\text{Intra}}\times \text{ df})\text{/}(\text{weight of sample},\text{ g})$$

### Determination of Biomass

The total fungal biomass was determined by measuring fresh weight per ml of fermentation medium after filtration through Whatman No. 1 filter paper, and the mycelia were washed two times with distilled water.

### Separation of Pigments for Application in the Preparation of Ice Lolly Products

The red, orange, and yellow pigments were separated and purified individually from the submerged culture as follows:

#### First: Separation of Red and Yellow Pigments Individually

**Figure 2:**
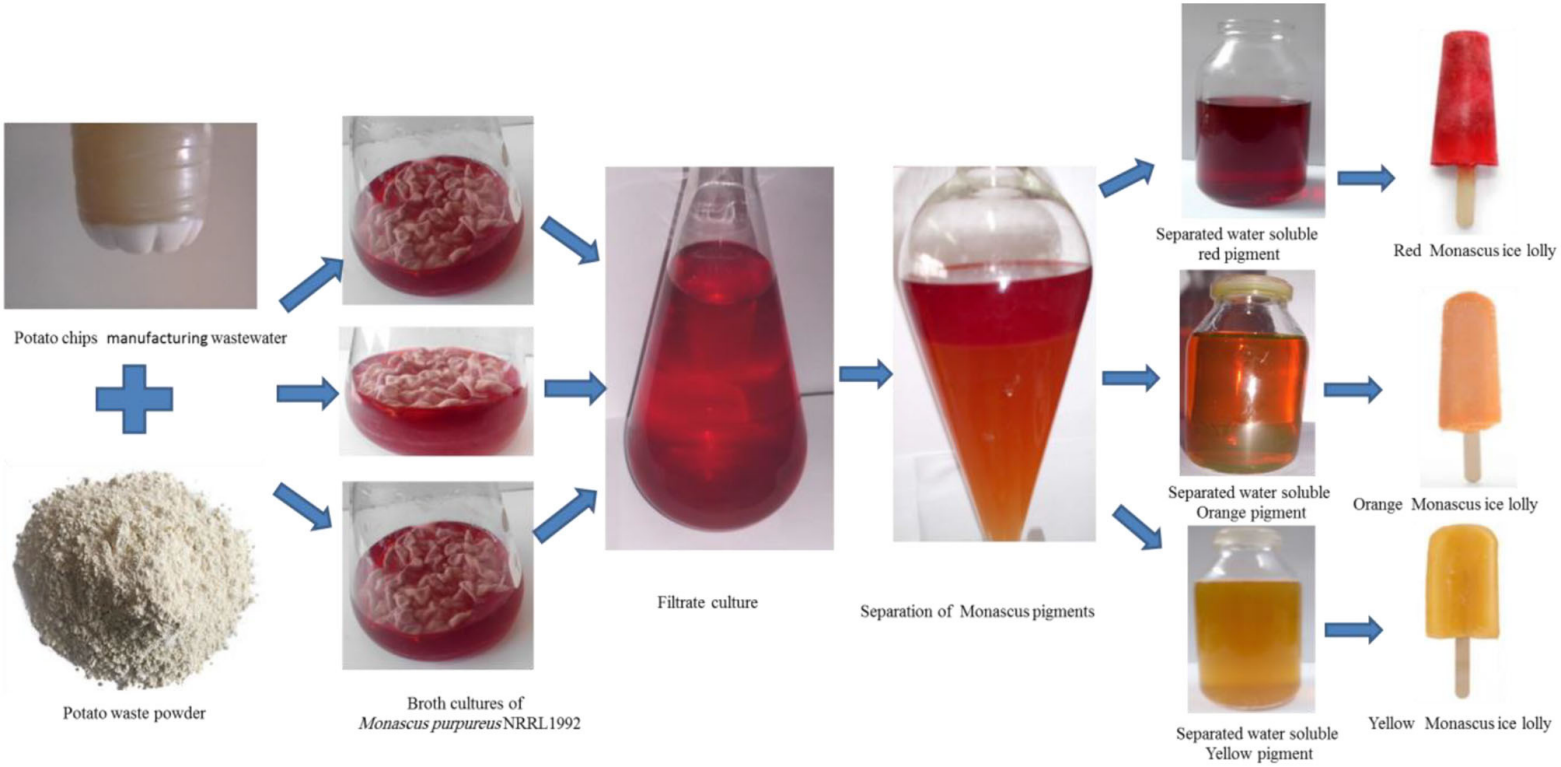
Production and separation of *Monascus purpureus* NRRL 1992 pigments and their applications in ice lolly.

The red and yellow pigments were extracted successively with ethyl acetate from culture filtrate (1:1 v/v), which was adjusted to pH 3.0 with 2 N HCl as shown in Figure 2. The ethyl acetate layer contains a mixture of red and yellow pigments. The ethyl acetate layer was eluted by a separating funnel. The ethyl acetate extract was evaporated in vacuo at 50°C. The semidry residue was dissolved in n-hexan and filtered through a filter paper (Whatman, No. 1; International Ltd., Maidstone, England) to separate the yellow pigment (the filtrate) from the red pigment (the residue). Separately yellow and red pigments were redissolving in a small amount of water by adjusting the pH to 7.0 with 2 N NaOH.

In addition, the yellow pigment can be separated from the ethyl acetate extract by evaporating the extract in vacuo at 50°C. The semidry residue was re-dissolved in a small amount of water by adjusting the pH to 7.0 with 2 N NaOH. Then 50 ml of cold acetone was added and kept at 4*C* for 30 min to precipitate yellow pigment. The acetone layer containing red pigment was collected, filtered, and concentrated further, and then taken in 20 ml of water. The yellow crystals, which settled at the bottom of the flask, were dissolved in 20 ml of water and kept in a glass vial.

#### Second: The Separation of Orange Pigments

The orange pigment, which eluted from the separation funnel, was collected, filtered, and concentrated further by evaporated in vacuo at 50*C*, and then taken in 20 ml of water separated water-soluble red, orange, and yellow pigments were used separately to prepare red, orange, and yellow ice lolly.

### Application of the Separated Pigments for the Coloring of Ice Lolly

The traditional methods manufactured ice lolly, and red, orange, and yellow pigments were added to the ingredients and then flavored with strawberry, orange, and banana, respectively. For ice lollies manufacturing, mix was standardized to sugar (20 %), stabilizer (0.5%), citric acid (0.3%), color (0.05%), flavor (0.45%), and water. Selecting ingredients, figuring the mix, blending, pasteurizing (68°C/30 min), transferring into molds, freezing (−18°C), and packing/storing until subjected to sensory evaluation (as described by Arbuckle, 1972).

Attributes of color, taste, odor, texture, and overall acceptability were tested using the slandered scorecard.

### Sensory Evaluation

A total of 10 trained panelists conducted sensory analyses of the samples (red, orange, and yellow ice lolly) according to the method described by Reitmeier and Nonnecke (1991). Attribute ratings were analyzed by analysis of variance and organoleptic tests (taste, color, texture, odor, and overall acceptability) using 10-point hedonic scales.

## Results and Discussion

### Chemical Composition Analysis

The wastewater used for experimental work was collected from the potato chips industry. The wastewater was obtained after the cutting (slicing) and soaking process, so this water contains a low microbial charge. The results obtained from the chemical analysis of the wastewater are presented in Table 2.

**Table 2:** Chemical composition of wastewater from potato chips manufacturing.

**pH**	**Carbohydrate (%)**	**Starch (%)**	**Reducing sugars (%)**	**Crude protein (%)**	**Crude fat (%)**	**Ash (%)**	**Zn (mg/L)**	**Ca (mg/L)**	**Mg (mg/L)**	**Fe (mg/L)**
7.30	4.94	1.96	0.01	1.50	0.11	0.14	6.50	280.00	245.00	45.5

The results show that the wastewater of cutting (slicing) and soaking from the potato chips manufacturing process contains a high amount of calcium and magnesium minerals and organic compounds of crude protein, starch, and carbohydrate. It also has a low content of crude fat, ash, reducing sugar, and zinc and iron minerals. As a result, these wastes could be considered valuable by-products if used as a fermentation medium to increase the value of the subsequent products and exceed the cost of reprocessing.

This study results are rather different from the study described by Gautam et al. (2017) who reported that wastewater of cutting (slicing) and soaking in potato chips industry contains 1.5% starch and it has a pH of 6.54.

### Pigment and Biomass Production

The results of BBD experimental design include 17 runs of 3 independent variables (potato wastes concentration, incubation period, and peptone concentration) for optimizing pigment production and biomass (dependent variables) by *M. purpureus* Went NRRL were listed in Table 3. The statistical test of total pigment and biomass produced was developed using analysis of variance (ANOVA) as shown in Tables 3, 4, respectively.

**Table 3:** A Box-Behnken experimental design of independent variables and actual results of response (1) total pigment and response (2) biomass produced by *Monascus purpureus* Went NRRL 1992.

	**Factor 1**	**Factor 2**	**Factor 3**	**Response 1**	**Response 2**
**Run**	**A: potato wastes concentration**	**B: fermentation period**	**C: peptone concentration**	**Total pigment**	**biomass**
	**%**	**days**	**%**	**AU/ml**	**g/ml**
1	7.5	10	3.5	13.61	0.11
2	7.5	10	3.5	15.024	0.12
3	10	10	5	10.28	0.213
4	7.5	10	3.5	16.15	0.12
5	5	10	2	44.33	0.168
6	7.5	7	5	16.64	0.081
7	7.5	10	3.5	20.26	0.114
8	10	10	2	25.94	0.17
9	7.5	13	2	48.89	0.12
10	5	13	3.5	29.48	0.128
11	10	13	3.5	17.61	0.262
12	7.5	13	5	22.84	0.11
13	10	7	3.5	16.68	0.144
14	7.5	7	2	47.7	0.046
15	7.5	10	3.5	16.072	0.098
16	5	7	3.5	22.21	0.123
17	5	10	5	10.31	0.132

**Table 4:** ANOVA for the entire quadratic model of response (1) concentration of total pigment.

**Source**	**Sum of squares**	**Mean square**	***F*** **-value**	***p*** **-value**	
Model	2440.89	271.21	59.88	<0.0001	Significant
A-potato wastes concentration	160.38	160.38	35.41	0.0006	
B-fermentation period	30.38	30.38	6.71	0.0359	
C-peptone concentration	1425.51	1425.51	314.75	<0.0001	
AB	10.05	10.05	2.22	0.1800	
AC	84.27	84.27	18.61	0.0035	
BC	6.28	6.28	1.39	0.2776	
A^2^	38.28	38.28	8.45	0.0227	
B^2^	289.17	289.17	63.85	<0.0001	
C^2^	380.57	380.57	84.03	<0.0001	
Residual	31.70	4.53			
Lack of fit	7.11	2.37	0.3857	0.7701	Not significant
Pure error	24.59	6.15			
Cor. total	2472.59				

Second-order quadratic model equation (Equation 1) (coded units) was used to express total pigment and biomass production as the following:


$$\begin{array}{l} {\text{Y}}_{\text{Pigment}}=16.22\;–\;4.48\text{A}+1.95\text{B }–13.35\text{C }–\;1.58\text{AB}\\ \;+\;1.59\text{AC}+1.25\text{BC}-3.02{\text{A}}^{2}+8.29{\text{B}}^{2}+9.51{\text{C}}^{2}\\ {\text{Y}}_{\text{Biomass}}=\;0.112\;+\;0.029\text{A }+\;0.028\text{B}+0.004\text{C }+\;0.028\text{AB}\\ \;+\text{ 0}.019\text{AC }–\;0.011\text{BC}+0.{\text{066A}}^{2}–\;0.{\text{014B}}^{2}–\;0.{\text{008C}}^{2} \end{array}$$

Y_Pigment_ is the total pigment concentration, Y_Biomass_ is the biomass weight g/ml, (A) potato wastes %, (B) fermentation period days, and (C) peptone concentration %.

**Figure 3:**
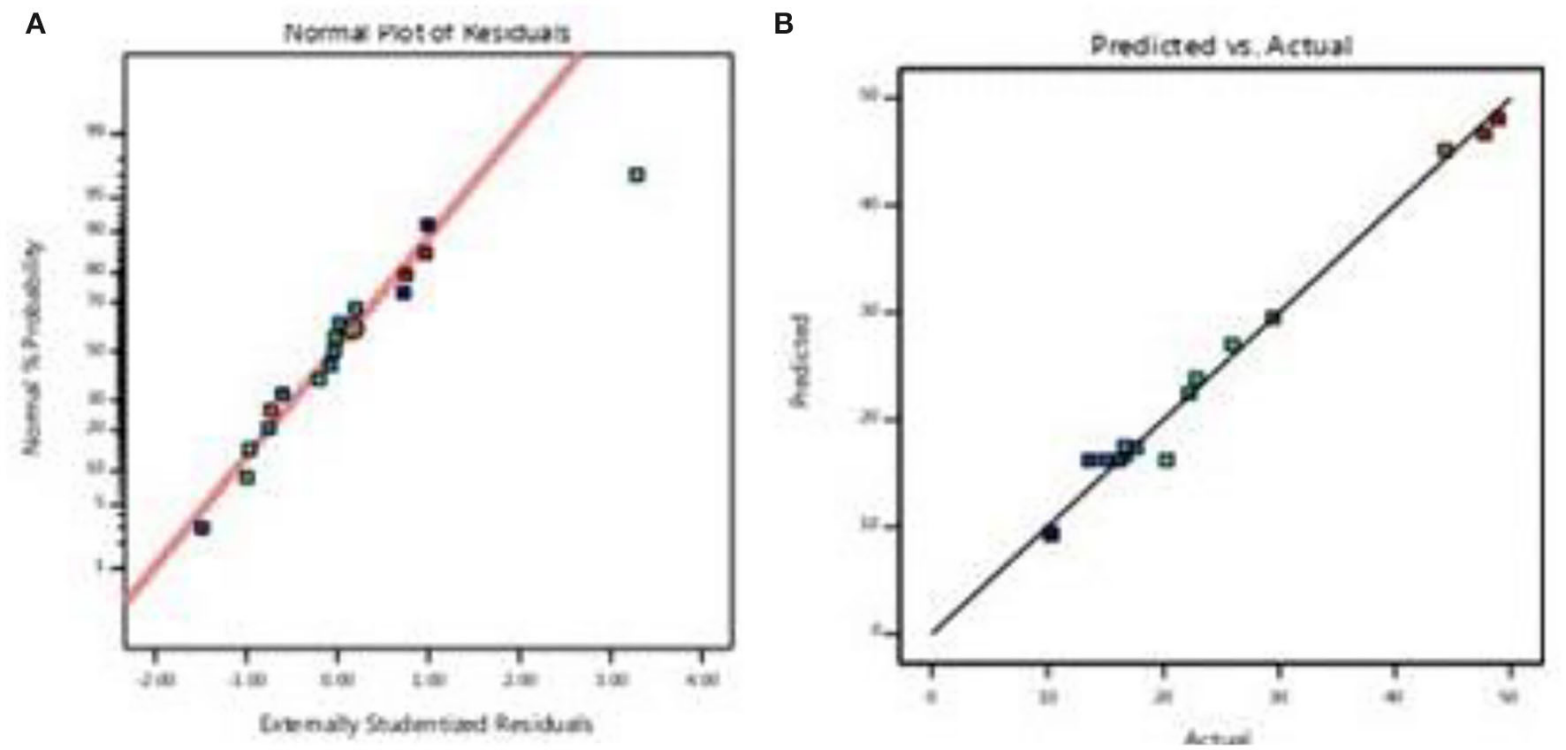
**(A)** Normal probability and **(B)** predicted vs. actual values.

In the case of optimization of total pigment production, the model's performance is usually indicated by the plots of the predicted vs. actual results (Figure 3B), which showed high correlation coefficients (R^2^ = 0.987), indicating that the predicted and actual values were in reasonable agreement. The correlation coefficient (*R*_2_) is a tool to check the “goodness of fit” between the experimental and the predicted results (Myers and Montgomery, 1995). In this case, *R*^2^ value of 0.9872 demonstrated that there is 98.7% of fitting between experimental and the predicted results, whereas the remaining percentage of 1.3% may have been due to other uncontrollable variables. The residual analysis was performed to investigate the suitability of the model. This was carried out by observing the normal probability plot of the residual in Figure 3A, where all the points lie on a straight line, suggesting that the errors were distributed evenly.

The adjusted coefficient of determination value *R*^2^adj is equal to 97%. This indicates that the regression equation provides a suitable model for the BBD experiment.

The adequate precision for total pigment is 23.77. SO, these quadratic models were significant for the process. The adequate precision test is used to determine the ratio of the predicted values at the design points to the average prediction error, when the ratio is more than 4; it means the model is acceptable and it can be used to explore the design space.

According to ANOVA (Table 4), the model obtained has a significant influence on the significance level (α) of 5% because of the high *F*-value (9.64) and low *p*-value (< 0.05). From Table 4, all independent variables (potato wastes concentration, incubation period, and peptone concentration) have a significant value (*p* < 0.05) for total pigment production. In addition, the value of the quadratic potato wastes concentration, incubation period, peptone concentration, and the interaction between potato wastes concentration and peptone concentration also showed a significant effect on the production of total pigment. Non-significant lack of fit means that the model is adequate to describe the observed data.

**Figure 4:**
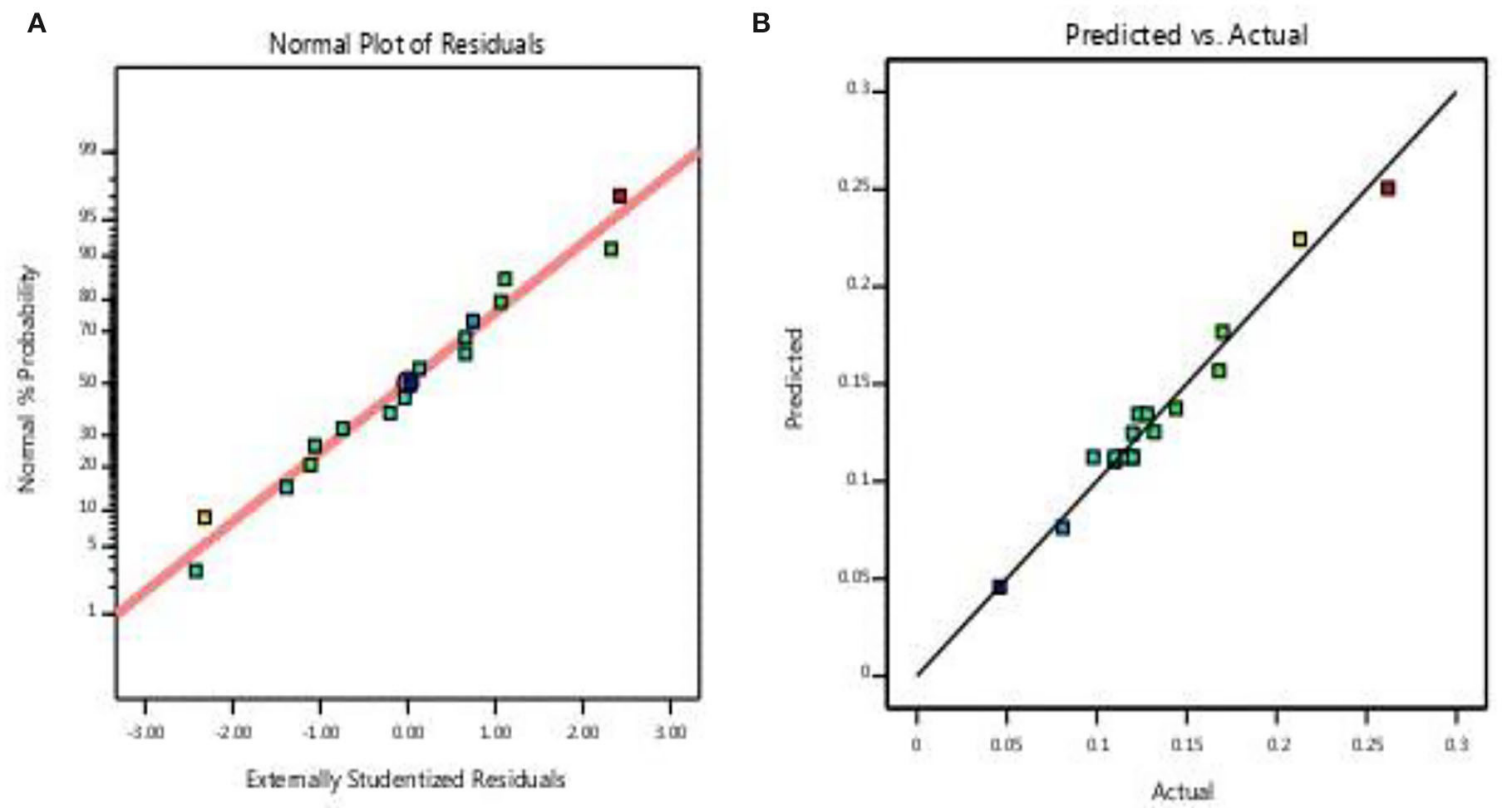
**(A)** Normal probability and **(B)** predicted vs. actual values.

In the case of biomass production, *R*^2^, *R*^2^adj, and Adeq precision were 0.972, 0.937, and 21.59, respectively. The normal probability plot indicated the quality of the adequacy of the current model where all residual points were on a straight line (Figure 4A). The predicted vs. actual plot of biomass experiment showed that the data were closely related, indicating that the model was highly fit (Figure 4B).

Based on ANOVA, which is listed in Table 5, the model of biomass was significant with a *p*-value (0.0001). The effect of potato wastes concentration and fermentation period was significant. Also, the interactions between potato wastes concentration and fermentation period and potato wastes concentration and peptone concentration were significant. The quadric effect of potato wastes concentration and fermentation period was significant.

**Table 5:** ANOVA for the entire quadratic model of response (2) concentration of biomass.

**Source**	**Sum of squares**	**Mean square**	***F*** **-value**	***p*** **-value**	
Model	0.0382	0.0042	27.80	0.0001	Significant
A-potato wastes concentration	0.0071	0.0071	46.33	0.0003	
B-fermentation period	0.0064	0.0064	41.78	0.0003	
C-peptone concentration	0.0001	0.0001	0.8376	0.3905	
AB	0.0032	0.0032	20.89	0.0026	
AC	0.0016	0.0016	10.21	0.0152	
BC	0.0005	0.0005	3.31	0.1115	
A^2^	0.0187	0.0187	122.49	<0.0001	
B^2^	0.0009	0.0009	6.06	0.0434	
C^2^	0.0003	0.0003	1.91	0.2095	
Residual	0.0011	0.0002			
Lack of fit	0.0007	0.0002	2.97	0.1600	Not significant
Pure error	0.0003	0.0001			
Cor. total	0.0393				

Total pigment concentration increased with increasing concentration of potato waste, and the maximum amount of total pigment was 48.89 AU/ml at 7.5% potato waste, while the maximum amount of biomass was 0.262 g/ml at 10% potato waste (Table 3); this is possible because potato waste was a suitable carbon source for pigment and biomass production, where the main role of carbon in fermentation medium was to produce cell biomass other than as an accelerator for the production of bioactive compounds through the secondary metabolism pathway (Table 6). De Carvalho et al. (2014) reported that some carbon sources that can be added include glucose, fructose, maltose, lactose, and galactose. Besides being able to increase pigment compounds and biomass, the right carbon source can produce high secondary metabolites. Also, starch is the main ingredient in rice, which is usually the major carbon source for *Monascus* growth and pigment production (Patakova, 2013; Chen et al., 2015). Abdel-Raheam et al. (2021) used potato chips manufacturing wastes as a suitable substrate for the production of pigment by *Monascus ruber* Went AUMC 5705 on solid-state fermentation. It was reported that the addition of a C source of 50 g/L can increase the growth rate of molds and increase the synthesis of pigment compounds, cell biomass, and ethanol production (Jones, 1998). By increasing the concentration of potato residues, more than 7.8% for the production of pigments, the productivity decreases, which could be explained by the accumulation of inhibitors and fermentation by-products in the fermentation medium. Wong et al. (1981) reported that high glucose concentration in the fermentation medium can be an advantage for mycelium growth, but as fermentation progresses, the fermentation medium becomes more acidic and this can lead to low pigment yields. But a further increase in glucose concentration progressively decreased the specific growth rate. This may have been an osmotic effect (Kim et al., 1997). Osmotic pressure increases with the increasing concentration of glucose and this reduces the water availability for microbial growth.

**Table 6:** Comparison between current study and others.

**Microorganism**	**Yield of pigment**	**Statistical analysis**	**References**	**Substrate**
*Monascus purpureus* (Went NRRL 1992)	Total pigment 29.86 AU/ml	(RSM)	Current study	Potato wastes
*Monascus purpureus* CMU001	Red pigment (22.25 UA/ml)	(RSM)	Silbir and Goksungur (2019)	Brewer's spent grain hydrolysate
*Monascus purpureus*	Total pigment 71.25 (CVU) color value unit	(RSM)	Dikshit and Tallapragada (2017)	Potato dextrose broth
*Monascus purpureus* (PTCC 5303)	Total pigment 4.38 (ODU/ml)	(RSM)	Seyedin et al. (2015)	Synthetic medium
*Monascus purpureus* (MTCC 369)	Total pigment 7.18 ODU/ml	(RSM)	Sharmila et al. (2013)	Potato powder
*Monascus anka* mutant	Yellow pigment 87.24 OD	(RSM)	Zhou et al. (2009)	Synthetic medium

The presence of nitrogen in molds growing medium is essential, where it is mainly included in primary metabolism, which is essential for microbial living and growth. It is also involved in secondary metabolism, which produced different bioactive compounds. Vidyalakshmi et al. (2009) reported that the addition of nitrogen can increase the metabolic activity of molds and the quantity of pigment compounds can be increased. In this study, peptone was used as a nitrogen source and it affected total pigment and biomass production positively, where the maximum amount of total pigment was 48.89 AU/ml at 2% peptone while 3.5% was the best for maximum biomass production of 0.262 g/ml (Table 3).

Celestino et al. (2014) reported that peptone provides many nutrients such as peptides and amino acids to the broth and it seems to be easily metabolized by most fungi, which can lead to increased production of their metabolites, including pigments. Also, they found that the use of nitrogen sources is based on the main substrate and other external factors, each of which also has an important role in the biosynthesis of bioactive compounds by *Monascus* sp.

Mousa et al. (2018) found that peptone was the most suitable nitrogen source among tested nitrogen sources (peptone, monosodium glutamate, yeast extract, sodium nitrate, and malt extract) for red pigment production by *M. purpureus* ATCC16436, and 2.5% of peptone was the best concentration for maximum pigment production. This result was near to our results; it was mentioned in many literatures that the nitrogen in peptone was available as amino acids, which help in the production of more stable extracellular pigment (Zhang et al., 2013; Ahmad and Panda, 2014). Also, peptone as an organic nitrogen source may be used as a carbon source. It may accelerate the protein-bound dissolution of red pigments into the culture broth (Broder and Koehler, 1980) and other previous studies (Table 6).

**Figure 5:**
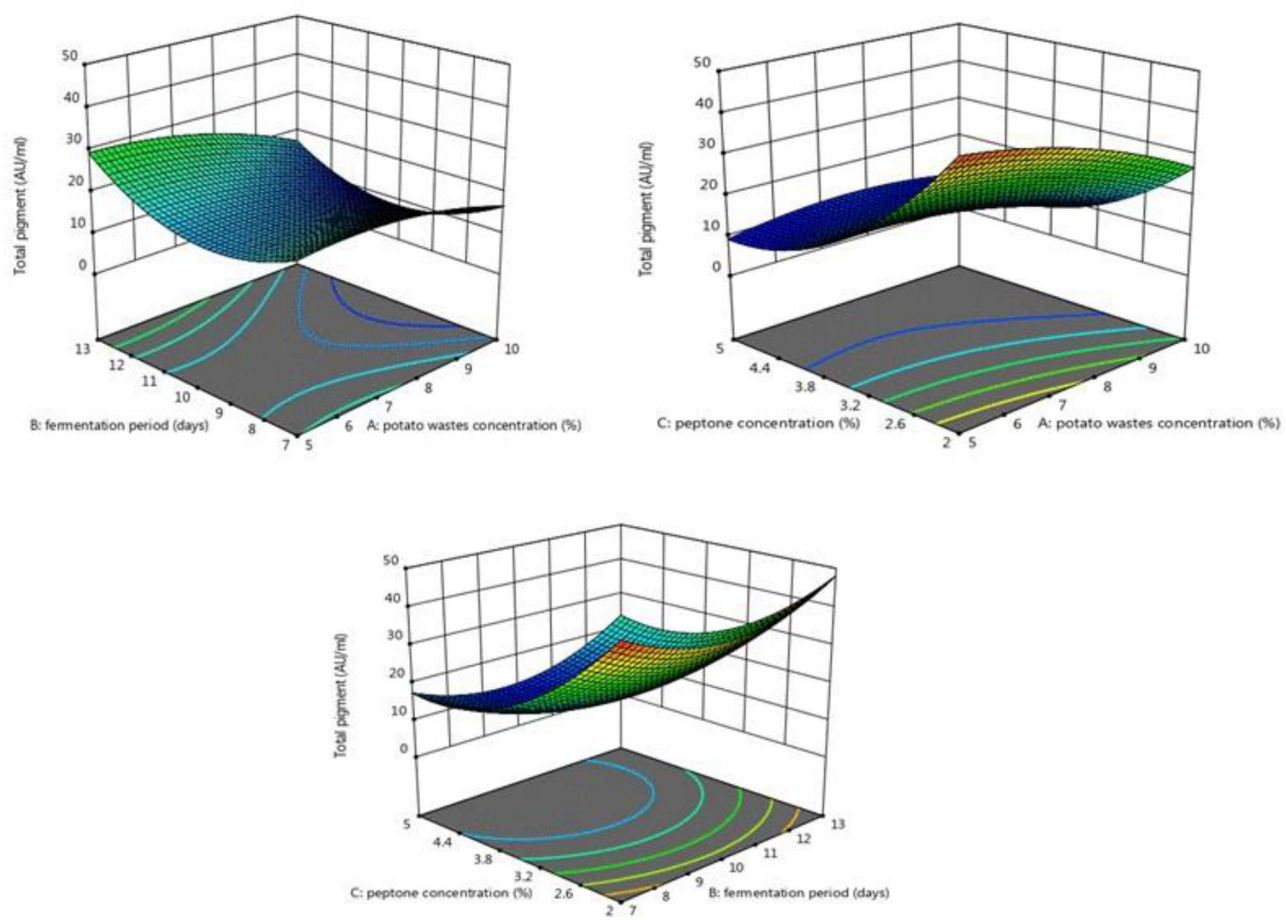
Response surface plots indicated the effect of potato wastes concentration, peptone concentration, and fermentation period on total pigment production.

Figure 5 shows that by increasing peptone concentration, the amount of pigment decrease. Mehri et al. (2021) found that the pigment production increased linearly up to 25 g/L of MSG concentration resulting in a maximum pigment synthesis of 45.7 UA_510nm_ and then declined at higher monosodium glutamate (MSG) concentrations. Raman et al. (2015) found that decreasing the concentration of nitrogen source in the production medium of melanin by *Aspergillus fumigatus* AFGRD105 has a positive effect on pigment production.

**Figure 6:**
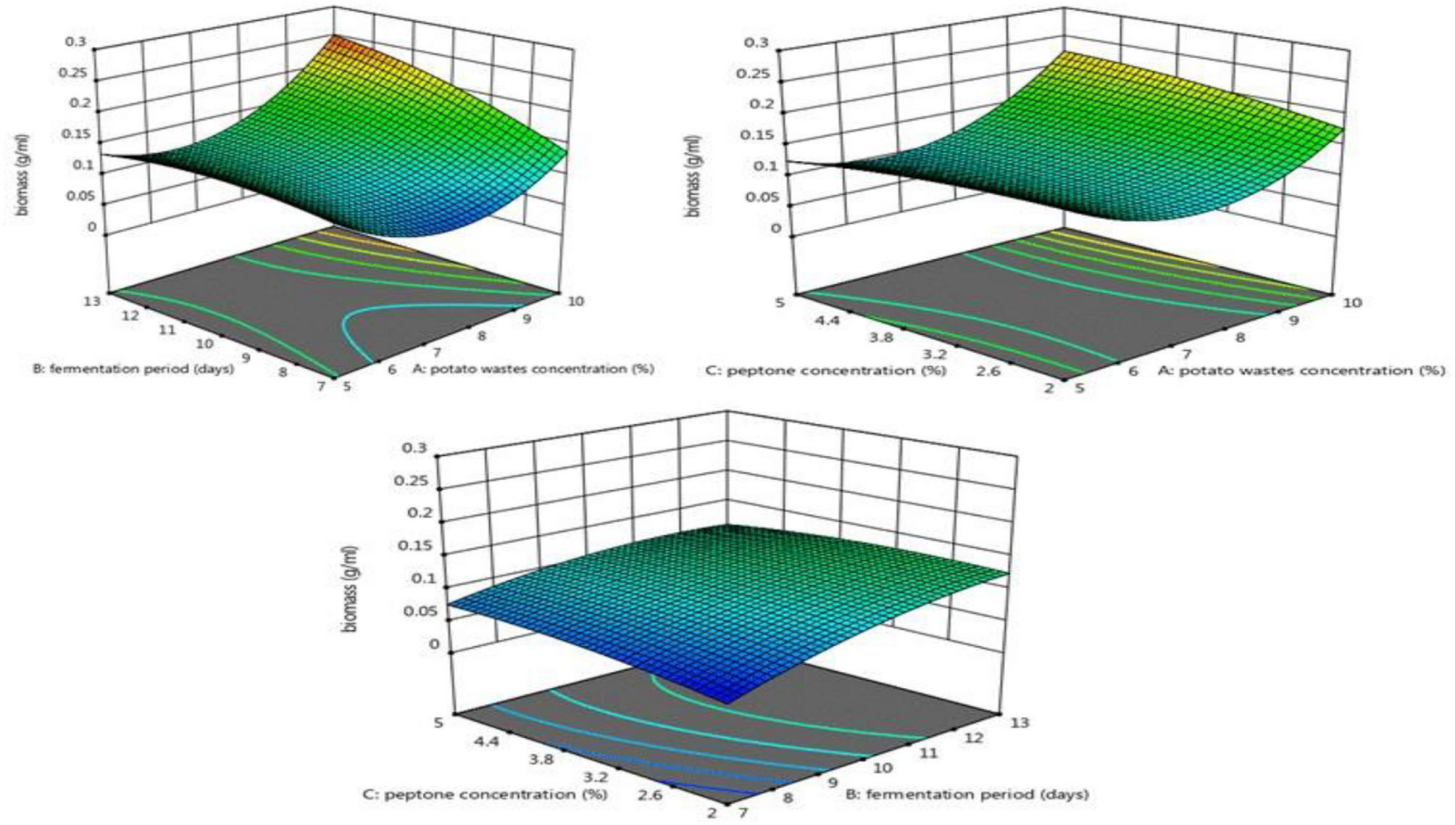
Response surface plots indicated the effect of potato wastes concentration, peptone concentration, and fermentation period on biomass production.

While Figure 6 shows the amount of biomass increase with increasing peptone concentration, this may be because of increasing peptone concentration C/N ratio will decrease, which has an effect on pigment and biomass production. Cho et al. (2002) mentioned that the C/N ratio usually affects the rates of biosynthesis of many metabolites, its influence on mycelia growth, and pigment production in fungi. Horwath (2007) demonstrated that 10–15 C/N ratio was preferred for biomass production, while more than 20 C/N ratio is preferred for pigment production. According to Ajdari et al. (2011), *M. purpureus* DSM1379 strain had the highest radial growth at C/N ratio of 1.8, while the lowest was obtained at C/N ratio of 10.03. On the contrary, *M. purpureus* FTC5357 strain had the highest radial growth at C/N ratio of 10.03, while the lowest was observed at C/N ratio of 1.8. They also concluded that the effect of C/N ratio on cell production was strain-dependent.

Fermentation of any substrate is influenced by the time of incubation. A significant increase in total pigment and biomass production of *M. purpureus* Went NRRL 1992 was observed by increasing fermentation period, which reached 12.8 days then they decreased. Sehrawat et al. (2017) found that variations in incubation time to achieve maximum pigment production may be due to strain specificity. Non-availability of nutrients might have ceased the growth of fungus. Johns et al. (1982) suggested that the reduction of pigment production by increasing the fermentation period may be due to pigments being degraded by an enzymatic pathway, which may be induced by nutrient exhaustion where enzyme degradation of secondary metabolites is a common phenomenon in fungi. Chen and Johns (1993) reported that a decrease in pigment production by *M. purpureus* was observed, which might be due to the decomposition of pigments (degradation of the chromophore pigment group or changes in the pigment structure). The incubation period in this study was shorter than that of which reported by Dikshit and Tallapragada (2011), Tallapragada et al. (2013), and Mousa et al. (2018) who reported that 16 days was the best incubation period for pigment production on broth medium by different *Monascus* sp. While Abdel-Raheam et al. (2019) mentioned that 11-day fermentation period gives the maximum amount of pigment by *M. ruber* Went AUMC 5705.

To test the validity of the model, an experiment was repeated in triplicate with optimized values of independent variables suggested by the model for maximum total pigment and a suitable amount of biomass: potato wastes concentration of 7.81%, fermentation period of 12.82 days, and peptone concentration of 2.87%. The amount of total pigment produced was 29.86 ± 1.42 AU/ml, which consisted of red, orange, and yellow pigments were 12.62 ± 0.61, 9.49 ± 0.47, and 7.75 ± 0.44 AU/ml, respectively, and the maximum biomass weight was 0.126 ± 0.003 g/ml, and the yield of pigment was 236.98 AU/g biomass, with 95.92% validity of the predicted model.

### Separation of the Produced Pigments

The pigment separation procedure utilized successfully resulted in the fractionation of crude *Monascus* pigments into three separate hues, such as red, orange, and yellow, ready for application as food colorants (Figure 2).

### Sensory Evaluations of the Produced Pigments as Colorant Additives for Ice Lolly

Pigments extracted and separated from submerged *M. purpureus* NRRL 1992 culture were used as colorant additives for ice lolly to enhance its appearance and acceptability. The red-, orange-, and yellow-flavored ice lollies were developed by individually adding the separated *Monascus* pigments. In this study, *M. purpureus* NRRL 1992 pigments were directly mixed with the food products during their preparation to impart red, orange, and yellow pigments individually to these products (Figure 2) and improved the aesthetic value.

The prepared ice lolly using *Monascus* pigments as colorants was sensory evaluated for taste, color, texture, odor, and overall acceptability by 10 panelists.

Data in Table 7 show the average sensory analysis scorecard and total scores for the separated *M. purpureus* NRRL 1992 pigments as natural colors for the butterscotch ice lolly. The overall average scores for the red, orange, and yellow ice lolly samples were 47.1, 46.4, and 46.5.

**Table 7:** Mean sensory scores of ice lolly samples colored with *Monascus purpureus* NRRL 1992 pigments.

**Items and score**	**Taste** **(10)**	**Color** **(10)**	**Odor** **(10)**	**Texture** **(10)**	**Overall acceptability** **(10)**	**Total score** **(50)**
**Name of product**						
Red ice lolly	9.2 ± 0.30	9.9 ± 0.10	9.3 ± 0.26	9.2 ± 0.10	9.5 ± 0.26	47.1 ± 0.3
Orange ice lolly	9.4 ± 0.2	8.8 ± 0.36	9.7 ± 0.26	9.5 ± 0.26	9 ± 0.26	46.4 ± 0.79
Yellow ice lolly	9.1 ± 0.46	9.6 ± 0.36	9.5 ± 0.44	9 ± 0.26	9.3 ± 5.29	46.5 ± 1.08

As indicated in Table 1, all produced food product samples colored with *M. purpureus* NRRL 1992 pigments received high ratings in all sensory assessed testing criteria (7).

The average scores for taste, color, texture, odor, and overall acceptability were between 8.8 and 9.9 scored as “like extremely” for all tested food products samples as described by Wang and Zhao (2008). The result showed that incorporating *M. purpureus* NRRL 1992 pigments for coloring prepared food products has proved to be excellent. The pigment is distributed evenly in the food product giving a pleasing appearance.

These results are in agreement with previous investigators who reported that food products gain more intense and stable color and improved organoleptic characteristics when *M. purpureus* pigments were used (Hamano and Kilikian, 2006 and Mal'a et al., 2010; Gomah et al., 2017; Abdel-Raheam et al., 2019, 2021; Darwesh et al., 2020).

Blanc et al. (1995) found that using *M. purpureus* pigment gave food items a more deep and persistent red hue and better organoleptic features. Furthermore, the use of natural pigments protects customers' health by lowering their salt intake and enables the production of totally natural foods without the use of synthetic chemicals (Su et al., 2005; Basuny and Abdel-Raheam, 2020). Traditionally, these pigments were used to make red rice, red wine, sausages, fish sauces, meat items, and soybean curd (Anonymous, 1999).

Vidyalakshmi et al. (2009) reported that *M. ruber* fermented rice (MFR) was employed as a colorant in the manufacturing of food items (Kesari) and had a very nice color and look. They also investigated the use of MFR for coloring flavored milk, which revealed that pigment dispersed uniformly, resulting in an attractive hue and acceptable look with higher acceptance.

## Conclusion

The utilization of potato waste powder is beneficial in manufacturing pigment from *M. purpureus* Went NRRL 1992. The BoxBehnken (BBD) design was influential in modeling total pigment production as a function of changes in process variables, potato waste concentration, fermentation period, and concentration of peptones. The optimum conditions were potato wastes concentration of 7.81%, fermentation period of 12.82 days, and peptone concentration of 2.87%, which produce total pigments of 29.86 AU/ml. The produced pigments were applied as coloring agents for ice lolly (red, orange, and yellow ice lolly). Applications of these natural colorants in the manufacturing of these products were judged to be extremely acceptable based on sensory assessment.

## Data Availability Statement

The original contributions presented in the study are included in the article/Supplementary Material, further inquiries can be directed to the corresponding author.

## Author Contributions

HA-R and MA: investigation, draft writing, and software and methodology. SG and AH: writing and reviewing. SA, MH and DM: reviewing. All authors contributed to the article and approved the submitted version.

## Conflict of Interest

The authors declare that the research was conducted in the absence of any commercial or financial relationships that could be construed as a potential conflict of interest.

## Publisher's Note

All claims expressed in this article are solely those of the authors and do not necessarily represent those of their affiliated organizations, or those of the publisher, the editors and the reviewers. Any product that may be evaluated in this article, or claim that may be made by its manufacturer, is not guaranteed or endorsed by the publisher.
