# Supplementary material for: Optimization of Monascus purpureus for Natural Food Pigments Production on Potato Wastes and Their Application in Ice Lolly
Source: Front Microbiol. 2022 Jun 1;13:862080. doi: 10.3389/fmicb.2022.862080 (PMC9199577; doi:10.3389/fmicb.2022.862080)
Supplement: Supplementary file 1 [file Table_1.DOCX]

**Supplementary data**

| Data prepared: | March 28, 2022 |
| --- | --- |
| Table: | **S1-S5** |
| Figure: | **S1-S2** |
| Pages: | **6** |

**Table S1:** A Box-Behnken experimental design of independent variables and actual results


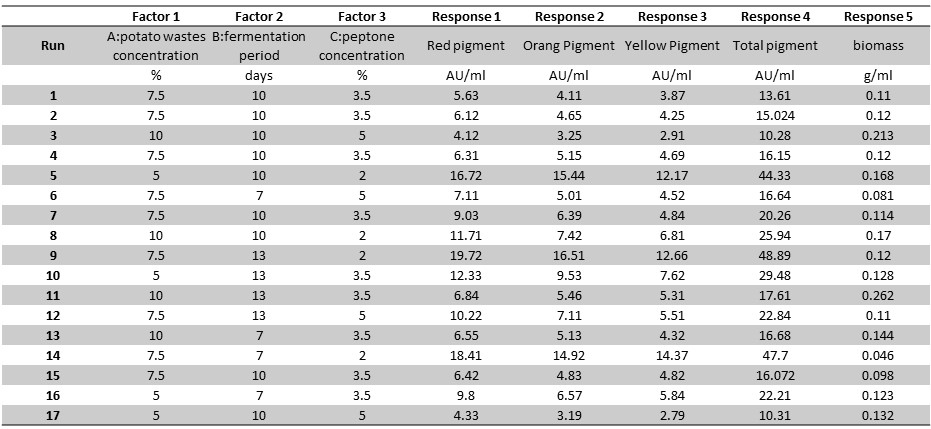


**Table S2:** (ANOVA) for Red pigment


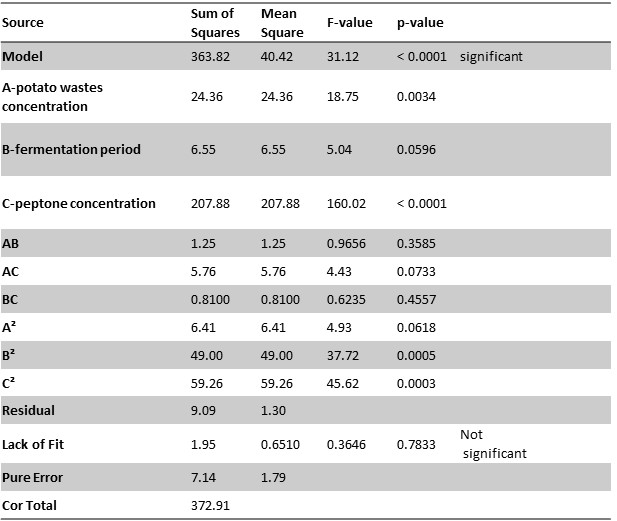


**Table S3:** (ANOVA) for Orang pigment


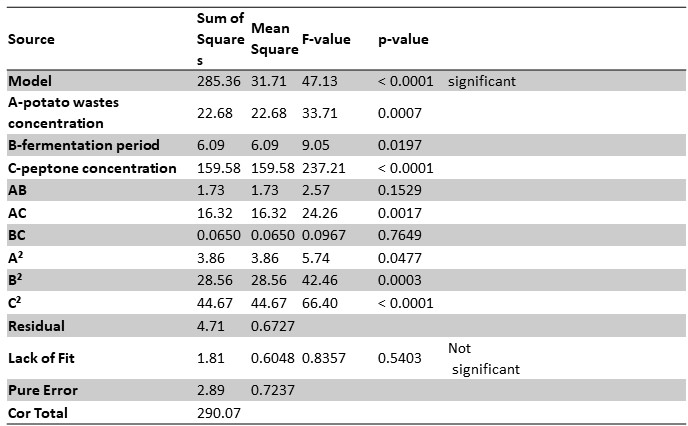


**Table S4:** (ANOVA) for Yellow pigment


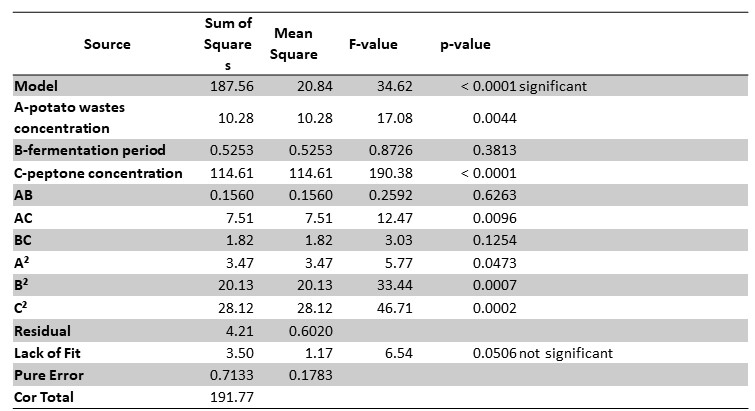


### Table S5: Fit Statistics

| **Color of pigment** | **Correlation coefficient R^2^ %** | **Adjusted R² %** | **Adeq Precision** |
| --- | --- | --- | --- |
| **Red pigment** | 97.56 | 94.43 | 18.1202 |
| **Orange pigment** | 98.38 | 96.29 | 21.5818 |
| **Yellow pigment** | 97.80 | 94.98 | 19.0224 |

Second-order quadratic model equation


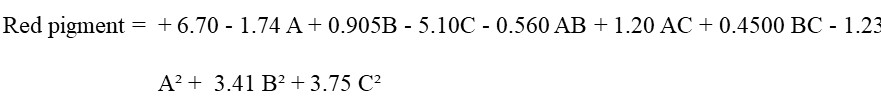


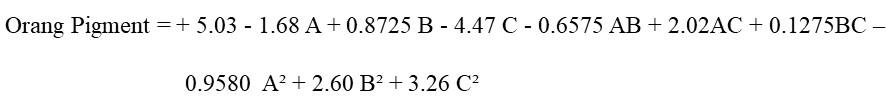


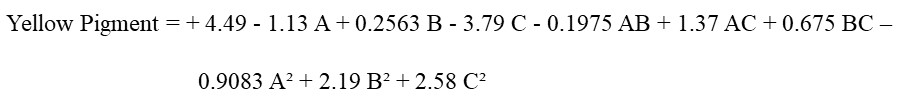


1. potato wastes %, (B) fermentation period days, (C) peptone concentration %.


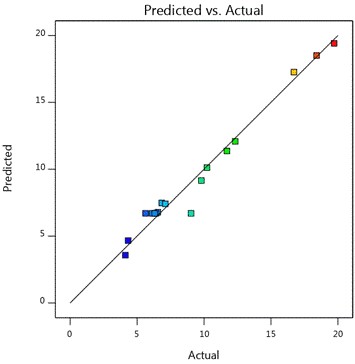

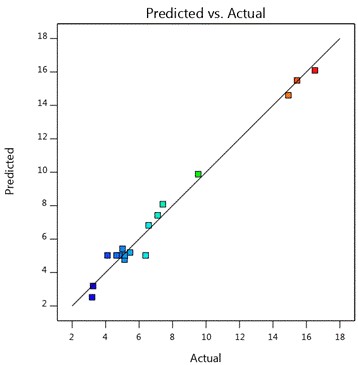

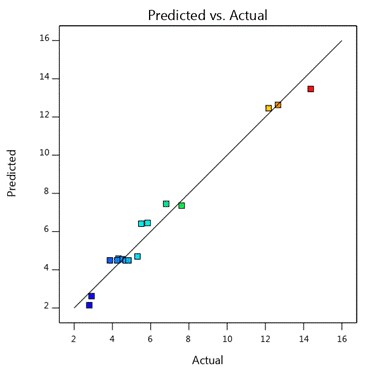


(c) Yellow pigment

(b) Orange pigment

1. Red pigment

**Figure S1:** predicted vs actual values of (a) Red, (b) Orange and (c) Yellow pigment


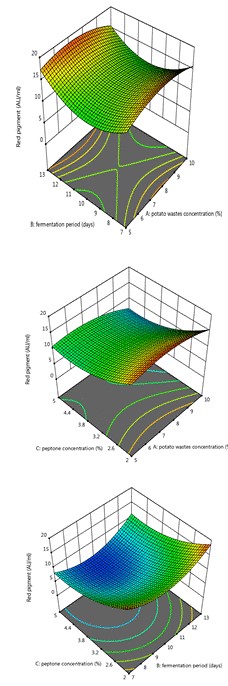

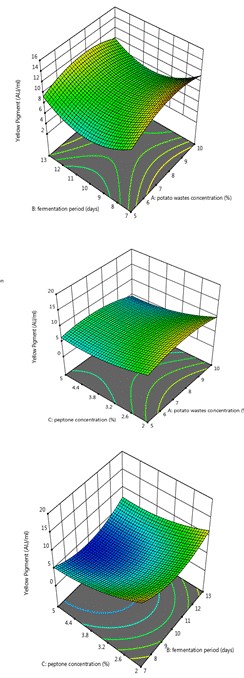

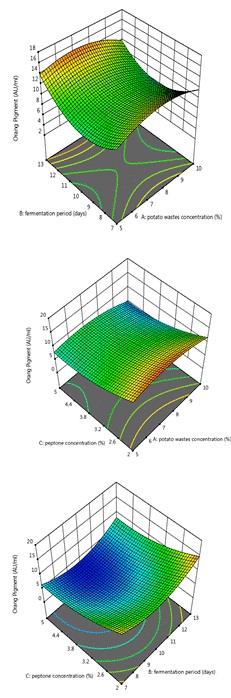


(c) Yellow pigment

(b) Orange pigment

1. Red pigment

**Figure S2:** Response surface plots indicated the effect of potato wastes concentration, peptone concentration and fermentation period on (a) Red, (b) Orange and (c) Yellow pigment
